# Primary Care for Gestational Diabetes: A Bibliometric Analysis of Publications from 1991 to 2024

**DOI:** 10.3390/ijerph21111405

**Published:** 2024-10-24

**Authors:** Aliya Makasheva, Lyudmila Yermukhanova, Khatimya Kudabayeva, Saule Tazhbenova, Maral Nogayeva, Aidana Tautanova, Aliya Zhylkybekova

**Affiliations:** 1 Department of Public Health and Health Care, West Kazakhstan Marat Ospanov Medical University, Aktobe 030012, Kazakhstan; yermukhanova@zkmu.kz (L.Y.); ds.oz@zkmu.kz (S.T.); 2 Department of Internal Diseases No. 1, West Kazakhstan Marat Ospanov Medical University, Aktobe 030012, Kazakhstan; h.kudabaeva@zkmu.kz; 3 Department of Rheumatology, S.D. Asfendiyarov Kazakh National Medical University, Tole-bi str. 94, Almaty 050000, Kazakhstan; nogaeva.m@kaznmu.kz; 4 Department of Microbiology, Virology named after Sh.I. Sarbasova, Astana Medical University, Beibitshilik str., 49a, Astana 010000, Kazakhstan; 202212537@amu.kz; 5 Department of Pathophysiology, West Kazakhstan Marat Ospanov Medical University, Aktobe 030012, Kazakhstan

**Keywords:** gestational diabetes, primary health care, bibliometric analysis

## Abstract

Gestational diabetes mellitus (GDM) represents a significant medical complication during pregnancy, with a global prevalence ranging from 2% to 26% and increasing by over 30% in recent decades. Therefore, the aim of our study is to assess the trends and distribution of published studies, as well as the contributions of countries, institutions, journals, and authors to the development of primary care for pregnant women with gestational diabetes. In this bibliometric analysis, we examine the role of primary health care in GDM from 1991 to 2024. The data were sourced from Scopus and Web of Science, encompassing 276 articles from 150 sources and involving 1375 authors. The analysis reveals a steady increase in publications, with a 4.29% annual growth rate. This study identifies the USA and UK as leading countries in GDM research, and there are significant international collaborations, with the USA having 17 joint articles with other countries. The University of Eastern Finland, Ohio State University, and Harvard University are noted as the most prolific institutions, with 23, 17, and 16 articles, respectively. Additionally, the journal *Diabetes Care* published the highest number of articles, totaling 635. Prominent authors such as Bernstein J. and McCloskey L., with seven articles each, have made substantial contributions to the field. Our work highlights the need to pay special attention to primary care for gestational diabetes, as many negative consequences of the disease can be prevented at this stage. Innovative approaches to screening for GDM can significantly improve treatment outcomes and reduce health risks, which will have long-term positive effects both for individual patients and society as a whole.

## 1. Introduction

According to the World Health Organization, GDM is hyperglycemia with blood glucose values above normal but below those diagnostic of diabetes and occurs during pregnancy [1]. GDM stands as a prevalent medical complication during pregnancy [2]. According to the International Diabetes Federation (IDF), hyperglycemia in pregnancy (HIP) affects approximately one in six pregnancies [3]. Globally, the prevalence of GDM ranges from 2% to 26%, and it has increased by more than 30% in the last few decades [4]. It is estimated that in 2021, 21.1 million (16.7%) live births came from women with some form of hyperglycemia during pregnancy. Of these cases, 80.3% were due to GDM, 10.6% were the result of diabetes diagnosed before pregnancy, and 9.1% were due to diabetes (including type 1 and type 2) first detected during pregnancy [3].

The major risk factors for the development of GDM in most countries include advanced maternal age during the first pregnancy [5], maternal obesity with a BMI of ≥30 kg/m^2^ [6], GDM in a previous pregnancy [7], ethnicity [8], pre-existing hypertension or cardiovascular disease [9], and a family history of diabetes or a prior large baby weighing ≥4000 g [10]. Moreover, in women who have had gestational diabetes, the probability of developing type 2 diabetes is several times higher than in women with normoglycemic pregnancies [11]. The development of GDM leads to complications such as the risk of developing gestational hypertension or cardiovascular diseases [9], the necessity of Caesarean section [12], obesity [13], and impaired carbohydrate metabolism, which can lead to the development of type 2 diabetes (T2DM) in both the mother and the child [14].

At the same time, in many low-, lower-middle-, and upper-middle-income countries, which account for over 85% of annual global deliveries, most women are either not screened or inadequately screened for diabetes during pregnancy [15]. Considering the link between hyperglycemia and poor pregnancy outcomes, there is a pressing need for a greater global emphasis on preventing, screening, diagnosing, and managing hyperglycemia during pregnancy [16,17]. If primary prevention measures are not implemented, the diabetes epidemic is poised to escalate, potentially emerging as one of the foremost contributors to disability and mortality worldwide if left unaddressed [18]. Therefore, early identification through screening, continuous monitoring, and comprehensive counseling on lifestyle and diet are essential components of primary care. Effective management and support from healthcare providers, particularly those involved in prenatal care, can significantly improve pregnancy outcomes and reduce the risk of long-term complications for both the mother and the child. Thus, primary care professionals are indispensable in the diagnosis, management, and guidance of pregnant women with GDM, ensuring a healthier future for both mothers and their babies.

While most existing bibliometric studies have focused on various aspects of GDM, our research emphasizes the role of primary health care in managing the condition. This area has been underexplored in previous analyses, highlighting the uniqueness and significance of our study. Furthermore, previous bibliometric analyses on GDM were conducted using CiteSpace and VOSviewer programs, and the authors included articles only up to 2020 from the Web of Science database [19,20,21,22]. Some of these articles focused on the connection between GDM and nutrition, and one study analyzed the 30 most cited articles in this field. In contrast, our study covers the period up to May 2024, using articles from both the Web of Science and Scopus databases. Additionally, we used the R-studio program (RStudio 2023.09.1+494, PBC, Boston, MA, USA) for data analysis in our research. Therefore, the aim of our study is to assess the trends and distribution of published studies, as well as the contributions of countries, institutions, journals, and authors to the development of primary care for pregnant women with gestational diabetes.

## 2. Materials and Methods

### 2.1. Search Strategy

**Figure 1:**
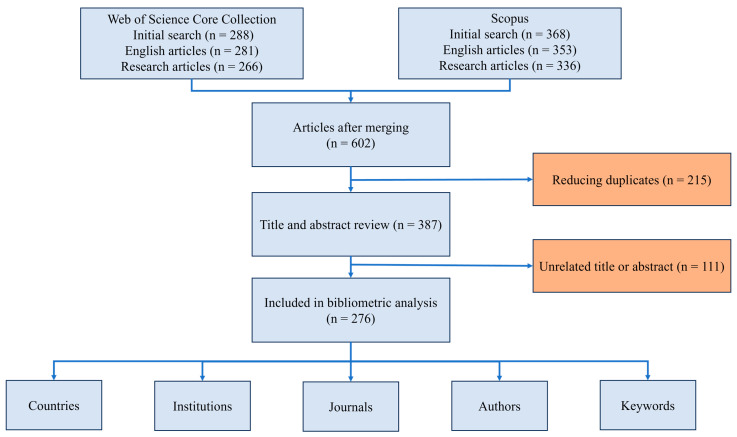
Search strategy.

In this study, we conducted a comprehensive search in the Scopus and Web of Science databases using advanced search capabilities. We utilized Boolean operators (AND, OR) to define keywords relevant to our research (Tables S1 and S2). Both original and review articles published in English were included in the study. The data were retrieved in BibTeX format for Scopus and TeX format for the Web of Science Core Collection [23]. Subsequently, the data were merged in RStudio (Studio 2023.09.1+494, PBC, Boston, MA, USA) into a single Excel file (Table S3). Articles considered irrelevant based on their titles, abstracts, and full-text content were systematically excluded. The search was conducted in May 2024, and the complete search strategy is presented in Figure 1.

### 2.2. Study Selection and Data Extraction

Study selection was conducted by two authors (A.M. and L.Y.). Independent article titles and abstracts identified through the search strategy were reviewed. Articles that were approved by both authors were subjected to a full-text analysis. If there was any disagreement, a third author provided the final decision.

### 2.3. Performance Analysis

Data management and bibliometric analysis were conducted using the Bibliometrix package (Version 4.1.3, Massimo Aria, University of Naples Federico II, Naples, Italy) and Biblioshiny web applications in RStudio (RStudio 2023.09.1+494, PBC, Boston, MA, USA) [24]. Most of the graphs were created using Biblioshiny Distribution (Version 4.1.3, Massimo Aria, University of Naples Federico II, Naples, Italy), while others were redrawn using Flourish.studio (Version N/A, Canva, London, UK).

## 3. Results

### 3.1. Summary of the Papers

A total of 276 relevant studies from 150 different sources were thoroughly analyzed. The analysis included materials from 1375 authors who collectively achieved an impressive average of 15.61 references per document over the past decade. In addition, the annual growth in this field of research was estimated to be 4.29%, which indicates a constant increase in the number of publications during the study period. The extensive scope of the research results was further emphasized by the inclusion of 5196 references and 532 unique author keywords

### 3.2. Trend of Publication

**Figure 2:**
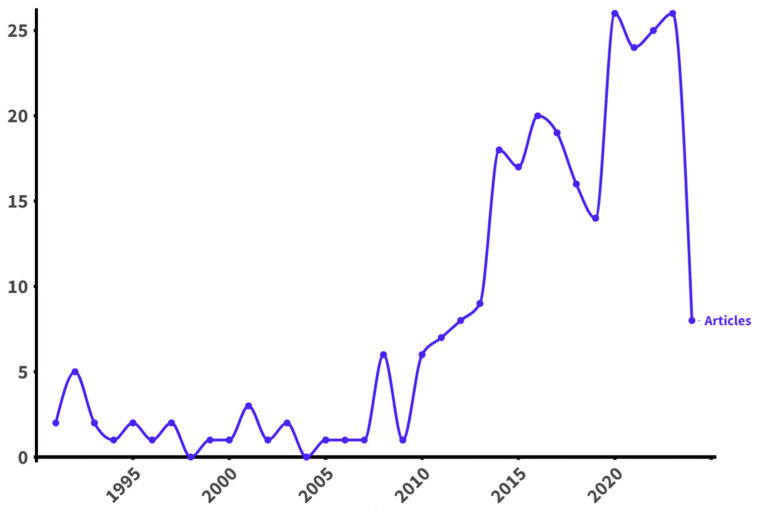
Global annual trend of publications on primary care for pregnant women with GDM (1991–2024).

Over the past three decades, there was a significant increase in the number of publications of scientific articles related to primary health care for pregnant women with GDM. In the period from 1991 to 2013, the fewest scientific publications were published, with only 63 studies. In contrast, the largest number of publications, a total of 131, were published between 2018 and 2023. Since 2010, we have seen an annual increase in the number of publications on this topic, except for the period from 2017 to 2019, which saw a minor decrease in activity. The peak of publication activity was in 2020 and 2023, when the number of publications was 26 in each year (Figure 2). The steady upward trend in publication activity is clearly visible in the graph, reflecting the growing research interest in this field year by year.

### 3.3. Countries, Institutions, and Their Collaboration Network

**Figure 3:**
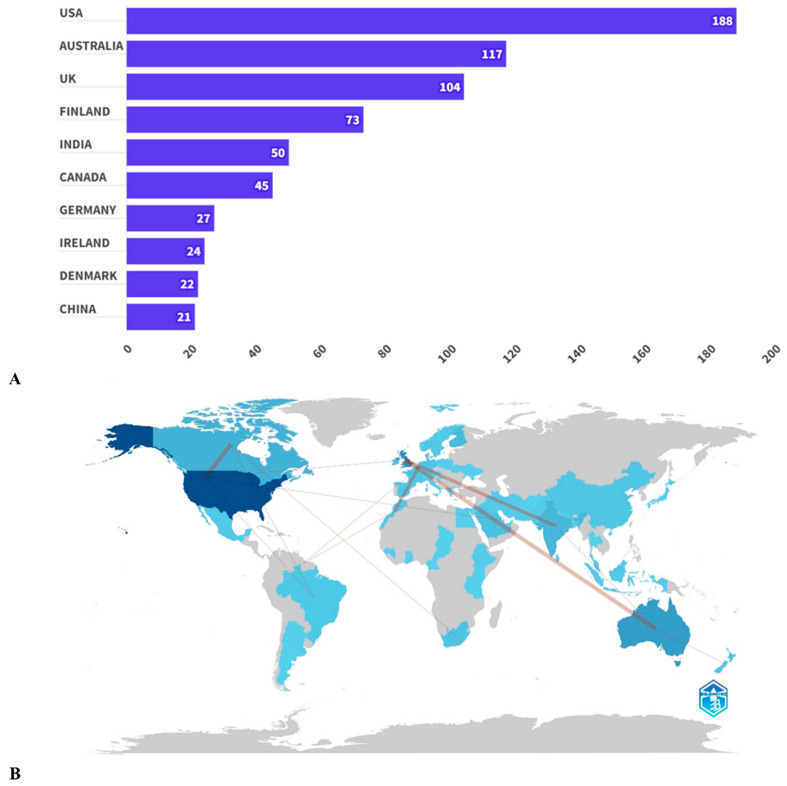
The top 10 countries with the highest number of scientific publications (**A**) and a world collaboration map (**B**). The color saturation intensity indicates the number of articles produced by each country, with darker shades representing higher publication volumes. The thickness of the connecting arrows illustrates the strength of collaboration between countries.

The United States, which has published 188 articles, ranks first in the study of primary care for gestational diabetes. It is followed by Australia with 117 and the United Kingdom with 104 articles (Figure 3A). It is noteworthy that the United States also shows the highest level of cooperation with other countries—17 articles, of which 6 are with Canada (Figure 3B). Australia has five joint projects with the UK, which indicates their close cooperation. In turn, the United Kingdom and India have four joint studies, as well as Belgium and Morocco. Table 1 lists the 10 leading organizations that have published the highest number of research and review articles on primary medical care for pregnant women with GDM from 1991 to 2024. The University of Eastern Finland leads with 23 articles, accounting for 31.5% of the total number of published works in this country. It is followed by Ohio State University with 17 articles and Harvard University with 16 articles. It is noteworthy that 50% of all universities included in this list are located in the United States.

### 3.4. Most Productive Journal

**Figure 4:**
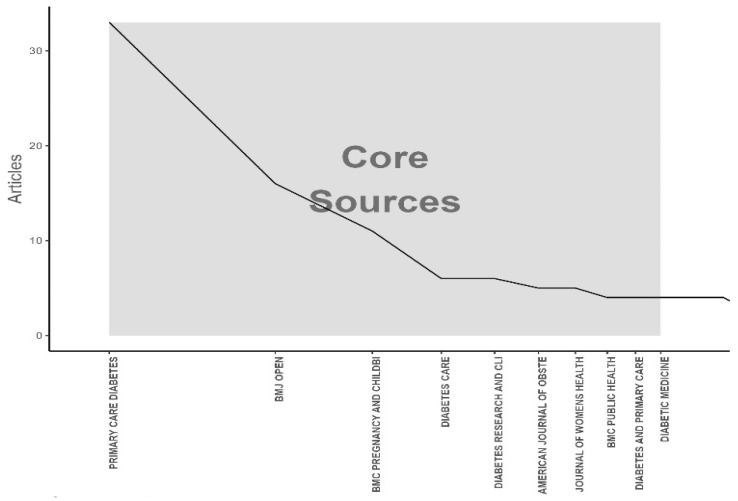
The plot of Bradford’s law identifies ten core journals on primary care for pregnant women with GDM (1991–2024).

Using Bradford’s law, we identified ten core journals that are the top choices for researchers publishing on primary care for pregnant women with GDM (Figure 4). Analyzing the publications revealed that the journal *Diabetes Care* with an impact factor of 16.2 was the most prolific, contributing 33 articles, which represents approximately 11.87%. *Lancet* and the *New England Journal of Medicine*, both publishing articles on this issue, have the highest impact factors of 168.9 and 158.5, respectively. It is noteworthy that 70% of these journals are in the upper quartile (Q1) of the *Endocrinology and Metabolism* and *Obstetrics and Gynecology* categories of the Extended Science Citation Index (SCIE) (Table 2).

### 3.5. Most Relevant Authors

**Figure 5:**
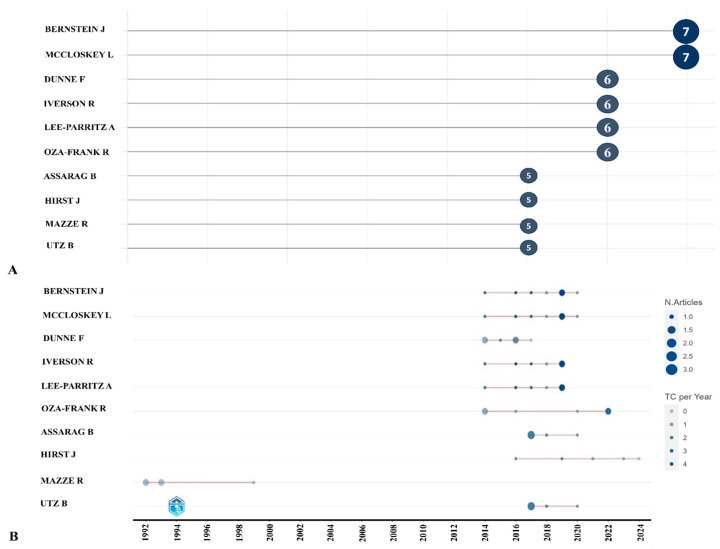
The top 10 most relevant authors (**A**) and their publication output (**B**) in 1991–2024.

Feig DS published the article “Risk of development of diabetes mellitus after diagnosis of gestational diabetes”, which had a total citation number of 327 in 2008 (Table 3). The most published authors are Bernstein J. and McCloskey L., who have seven articles on this research area (Figure 5A). Bernstein J., Mccloskey L., Iverson R., and Lee-Parritz A. each published two articles with total citations per year of 4.33 in 2019 (Figure 5B). It is noteworthy that all four authors were co-authors in one article published in 2019. One of the first to research the topic of primary care for GDM was Mazze R, who published his articles between 1992 and 1999.

### 3.6. Key Words

**Figure 6:**
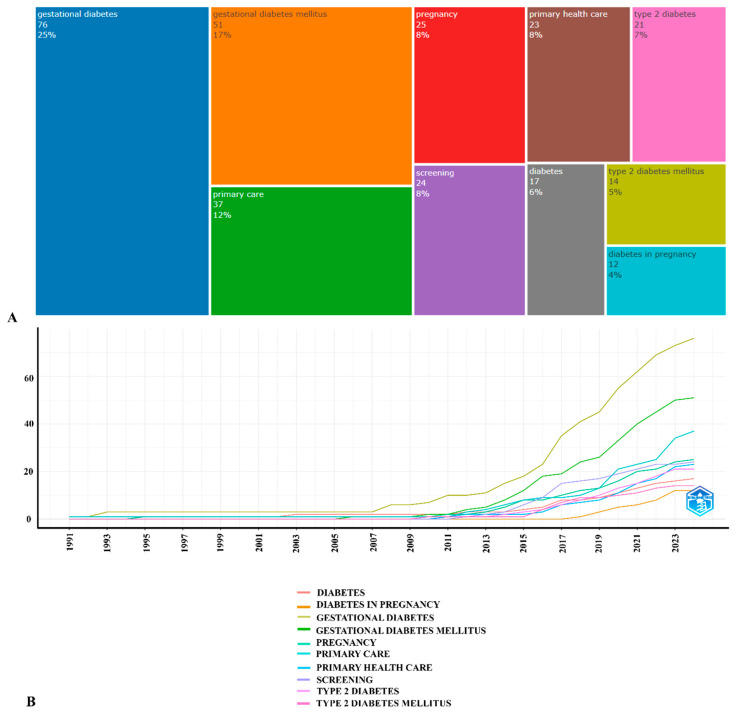
TreeMap (**A**) and scatter plot (**B**) representing top ten author keywords in research on primary care for pregnant women with GDM (1991–2024).

Figure 6A shows the top 10 author keywords. “Gestational diabetes” accounts for 25% of the total, followed by “primary care” and “pregnancy” accounting for 12% and 9%, respectively, indicating their significant roles in diabetes-related studies. Since the early 1990s and until 2007, these terms were mentioned to a lower degree. However, a noticeable increase began around 2008, with terms like “gestational diabetes”, “gestational diabetes mellitus”, and “primary care”. By 2023, these terms had the highest cumulative occurrences, indicating a growing focus on diabetes during pregnancy within the research community. Other terms such as “screening” and “primary health care” also showed substantial growth, reflecting an increased interest in preventive and primary healthcare measures in diabetes management (Figure 6B).

## 4. Discussion

This bibliometric analysis aims to illustrate the scope and characteristics of the scientific literature on primary care in GDM. This study covered a period in the last three decades, paying special attention to the indicators of publication activity and citations, as well as the most frequently used author keywords. Based on the frequency of publications and their citations, we identified the top ten relevant authors, the most cited documents, and the leading journals in the field. In addition, the interaction between well-known institutions and countries that have contributed to research in the field of primary care for pregnant women with GDM in recent years was considered.

In recent years, the volume of scientific research on this topic has increased significantly, as confirmed by another bibliometric study conducted by Tantengco OAG on GDM trends in Southeast Asia [20]. This upward trend indicates the increased interest of researchers in primary care in GDM. A growing volume of research highlights the urgent need to address the issues associated with postpartum screening in women who have had GDM [35,36]. These issues may be linked to problems in the diagnosis of GDM and the lack of uniform standards, resulting in significant variation in the prevalence of GDM across different countries [37]. The presence of GDM may subsequently lead to side outcomes such as the development of type 2 diabetes in both the mother and child.

The field of GDM is actively evolving and gaining interest both nationally and internationally. The United States, Canada, Australia, the United Kingdom, and Finland have become leading countries in GDM research, reflected in their high levels of publications. These data are consistent with the findings of another bibliometric analysis conducted by Iftikhar PM in 2019 [19]. High publication activity in developed countries may indicate that they have more opportunities to research this issue, and their healthcare systems pay close attention to the screening and prevention of GDM. One example is the National Academy of Sciences (NAS), founded in America in 1863 [38]. This organization, with the help of funding, develops science in its country, giving researchers the opportunity to study problems in their field in detail.

Collaboration provides opportunities to expand research in this field and to study the characteristics of this disease in more detail, depending on the place of residence, nationality, lifestyle, and other factors [39,40,41]. Joint work increases their recognition at the international level, reveals the problem from different points of view, and provides an opportunity to share experiences. Institutions such as the University of Eastern Finland and Ohio State University were identified as major contributors to the field. The University of Eastern Finland conducted a notable study in 2014 across five hospital districts focusing on lifestyle intervention and the prevention of type 2 diabetes in pregnant women with GDM [42]. Meanwhile, Ohio State University examined postpartum follow-up schemes for patients after the diagnosis of GDM [43]. The high number of publications and citations from these institutions highlights their influential role in advancing research on primary care for GDM.

Journals included in core sources in the field of GDM comprise publications specializing in endocrinology, metabolism, obstetrics, gynecology, general medicine, and internal medicine. Authors typically select journals based on the journal’s impact, quartile, and reputation. High-impact journals are preferred because they are more likely to disseminate research findings widely and influence clinical practice guidelines and health policy decisions [44]. Choosing the right journal is crucial for policymakers in health care to obtain reliable information. Consequently, the strategic selection of journals by researchers not only enhances the visibility and impact of their work but also ensures that the findings reach key stakeholders who can implement evidence-based improvements in GDM management [45,46].

The research of Bernstein J., McCluskey L., and Randy Iverson Lee-Paris focuses on the prevention of early development of type 2 diabetes mellitus after pregnancies complicated by GDM [47,48]. Their joint paper points out that the recommended 2 h oral glucose tolerance test is too difficult for women and healthcare providers to perform as prescribed [49]. This leads to low glucose testing rates and a high risk of developing diabetes mellitus, which reaches 20% [25]. Despite having insurance coverage, many women do not seek medical help, which may be due to a barrier in communication between pregnant women and medical professionals. This indicates the need to take measures to reduce the gap between obstetrics and primary health care in order to ensure preventive monitoring and timely detection of GDM [50].

An analysis of the cumulative occurrence of the authors’ keywords related to diabetes over time provides valuable information about research trends in this area. Since 2008, there has been a rapid increase in the use of these terms, and more emphasis is being placed on the prevention and diagnosis of this disease. Primary care for GDM is becoming increasingly important, as attention to the prevention and screening of this condition increases every year. The introduction of new screening programs that will more effectively identify GDM is necessary for timely intervention. Untimely detection and treatment of GDM can lead to the development of type 2 diabetes in women after childbirth.

Our bibliometric analysis reveals a significant upward trend in publications related to GDM, indicating a growing interest in the field. Additionally, the contributions of prominent authors and institutions underscore their pivotal role in advancing both research and clinical practice in primary care for pregnant women with GDM.

### The Limitations of this Study

Our bibliometric analysis provides valuable insights into the role of primary care for pregnant women with GDM, yet it has several limitations. Firstly, we relied on the Scopus and WOS-CC databases, which may lead to a partial representation of the global literature by excluding other databases. However, despite this, we integrated two databases, a feature absent in other studies focused on GDM, thereby enhancing the comprehensiveness of our review.

Secondly, by including only English-language articles, we may have introduced language bias, potentially excluding relevant studies in other languages. Despite these limitations, our analysis significantly contributes to understanding the scholarly landscape of primary care for pregnant women with GDM. Researchers should consider these constraints and leverage our findings to guide further investigations in this field.

## 5. Conclusions

GDM poses a serious threat to maternal and child health, especially given the increasing global prevalence of diabetes. Given the growing global prevalence of diabetes mellitus, changes in health policy are needed to develop new, more effective screening and education models. Modern GD screening models often turn out to be ineffective due to insufficient compliance with standards and the difficult timing of glucose testing by both pregnant women and medical professionals. Our work highlights the need to pay special attention to primary care for gestational diabetes, as many negative consequences of the disease can be prevented at this stage. Innovative approaches to screening for GDM can significantly improve treatment outcomes and reduce health risks, which will have long-term positive effects both for individual patients and for society as a whole. This study provides a comprehensive overview of the trends and distribution of research on GDM, highlighting the crucial contributions of key authors and institutions. These findings emphasize the importance of collaborative research efforts in improving primary care outcomes for women with gestational diabetes.

## Figures and Tables

**Table 1:** Most relevant affiliations.

Rank	Affiliation	Articles	Country
1	University of Eastern Finland	23	Finland
2	Ohio State University	17	USA
3	Harvard University	16	USA
4	Ollscoil Na Gaillimhe-University of Galway	14	Ireland
5	Kuopio University Hospital	13	Finland
6	Boston University	12	USA
7	University of Oxford	12	UK
8	Johns Hopkins University	11	USA
9	University System of Ohio	11	USA
10	University of London	10	UK

**Table 2:** The top ten most cited journals on primary care for pregnant women with GDM (1991–2024).

Rank	Sources	Articles	IF	JCR Category (Quartile)
1	*Diabetes Care*	635	16.2	Endocrinology and Metabolism—SCIE (Q1)
2	*Obstetrics and Gynecology*	225	7.2	Obstetrics and Gynecology—SCIE (Q1)
3	*American Journal of Obstetrics and Gynecology*	205	9.8	Obstetrics and Gynecology—SCIE (Q1)
4	*Diabetic Medicine*	162	3.5	Endocrinology and Metabolism—SCIE (Q3)
5	*Diabetes Research and Clinical Practice*	154	5.1	Endocrinology and Metabolism—SCIE (Q2)
6	*Lancet*	141	168.9	Medicine, General and Internal—SCIE (Q1)
7	*New England Journal of Medicine*	138	158.5	Medicine, General and Internal—SCIE (Q1)
8	*BMC Pregnancy and Childbirth*	119	3.1	Obstetrics and Gynecology—SCIE (Q1)
9	*PLoS ONE*	87	3.7	Multidisciplinary Sciences—SCIE (Q2)
10	*Diabetologia*	86	8.2	Endocrinology and Metabolism—SCIE (Q1)

**Table 3:** The ranking of the 10 most globally cited documents on primary care for pregnant women with gestational diabetes.

Rank	Study References	Title of the Document	Journal Name	DOI	Total Citations
1	Feig D.S., 2008 [25]	Risk of development of diabetes mellitus after diagnosis of gestational diabetes	*Can. Med. Assoc. J.*	https://doi.org/10.1503/cmaj.080012	327
2	Daly B., 2018 [26]	Increased risk of ischemic heart disease hypertension and type 2 diabetes in women with previous gestational diabetes mellitus a target group in general practice for preventive interventions a population-based cohort study	*PLoS Med.*	https://doi.org/10.1371/journal.pmed.1002488	182
3	Bennett W.l., 2014 [27]	Utilization of primary and obstetric care after medically complicated pregnancies an analysis of medical claims data	*J. Gen. Intern. Med.*	https://doi.org/10.1007/s11606-013-2744-2	135
4	Carson M.P., 2013 [28]	Original research postpartum testing rates among women with a history of gestational diabetes systematic review	*Prim. Care Diabetes*	https://doi.org/10.1016/j.pcd.2013.04.007	86
5	Leiter L.A., 2001 [29]	Diabetes screening in canada diascan study prevalence of undiagnosed diabetes and glucose intolerance in family physician offices	*Diabetes Care*	https://doi.org/10.2337/diacare.24.6.1038	80
6	Almario C.V., 2008 [30]	Obstetricians seldom provide postpartum diabetes screening for women with gestational diabetes	*Am. J. Obstet. Gynecol.*	https://doi.org/10.1016/j.ajog.2007.11.001	76
7	Coton S.J., 2016 [31]	A cohort study of trends in the prevalence of pregestational diabetes in pregnancy recorded in uk general practice between 1995 and 2012	*BMJ Open*	https://doi.org/10.1136/bmjopen-2015-009494	76
8	Barbour L.A., 2018 [32]	A cautionary response to SMFM statement pharmacological treatment of gestational diabetes	*Am. J. Obstet. Gynecol.*	https://doi.org/10.1016/j.ajog.2018.06.013	73
9	Gabbe S.G., 2012 [33]	Promoting health after gestational diabetes a national diabetes education program call to action	*Obstet. Gynecol.*	https://doi.org/10.1097/AOG.0b013e3182393208	70
10	Rumbold A.R., 2011 [34]	Delivery of maternal health care in indigenous primary care services baseline data for an ongoing quality improvement initiative	*BMS Pregnancy Childb.*	https://doi.org/10.1186/1471-2393-11-16	68

## Data Availability

The data are contained within the article.

## Supplementary Materials

The following supporting information can be downloaded at: https://www.mdpi.com/article/10.3390/ijerph21111405/s1, Supplementary Table S1: The R code utilized for integrating data from the Web of Science and Scopus databases. This code imports datasets from both sources, merges them, eliminates duplicates, and exports the consolidated data into an Excel file. Supplementary Table S2 and Table S3: The search queries used to retrieve relevant articles from the Scopus and Web of Science databases, respectively.
