# Supplementary material for: Primary Care for Gestational Diabetes: A Bibliometric Analysis of Publications from 1991 to 2024
Source: Int J Environ Res Public Health. 2024 Oct 24;21(11):1405. doi: 10.3390/ijerph21111405 (PMC11594181; doi:10.3390/ijerph21111405)
Supplement: Supplementary file 1 [file ijerph-21-01405-s001.zip › ijerph-3122665-supplementary.pdf]

Table S1. Scopus database.

Query
(TITLE-ABS-KEY (“Diabetes, Gestational” OR “Diabetes, Pregnancy-Induced” OR “Diabetes, Pregnancy Induced” OR “Pregnancy-Induced Diabetes” OR “Gestational Diabetes” OR “Diabetes Mellitus, Gestational” OR “Gestational Diabetes Mellitus”) AND TITLE-ABS-KEY (“Primary Health Care” OR “Care, Primary Health” OR “Health Care, Primary” OR “Primary Healthcare” OR “Healthcare, Primary” OR “Primary Care” OR “Care, Primary”) AND (LIMIT-TO (LANGUAGE , "English"))

Table S2. Web of Science database

No	Queries
#1	“Diabetes, Gestational” OR “Diabetes, Pregnancy-Induced” OR “Diabetes, Pregnancy Induced” OR “Pregnancy-Induced Diabetes” OR “Gestational Diabetes” OR “Diabetes Mellitus, Gestational” OR “Gestational Diabetes Mellitus” (Title) or “Diabetes, Gestational” OR “Diabetes, Pregnancy-Induced” OR “Diabetes, Pregnancy Induced” OR “Pregnancy-Induced Diabetes” OR “Gestational Diabetes” OR “Diabetes Mellitus, Gestational” OR “Gestational Diabetes Mellitus” (Author Keywords) “Diabetes, Gestational” OR “Diabetes, Pregnancy-Induced” OR “Diabetes, Pregnancy Induced” OR “Pregnancy-Induced Diabetes” OR “Gestational Diabetes” OR “Diabetes Mellitus, Gestational” OR “Gestational Diabetes Mellitus” (Abstract)
#2	“Primary Health Care” OR “Care, Primary Health” OR “Health Care, Primary” OR “Primary Healthcare” OR “Healthcare, Primary” OR “Primary Care” OR “Care, Primary” (Title) or “Primary Health Care” OR “Care, Primary Health” OR “Health Care, Primary” OR “Primary Healthcare” OR “Healthcare, Primary” OR “Primary Care” OR “Care, Primary” (Author Keywords) or “Primary Health Care” OR “Care, Primary Health” OR “Health Care, Primary” OR “Primary Healthcare” OR “Healthcare, Primary” OR “Primary Care” OR “Care, Primary” (Abstract)
#3	#1 and #2

Table S3. Codes were used to merge Scopus and Web of Science exported data in RStudio

```
library(bibliometrix)
library(openxlsx)
## importing web of science dataset
web_data<-convert2df("abs.txt")
## importing scopus dataset
scopus_data<-convert2df("abs.bib",dbsource="scopus",format="bibtex")
##combined both datasets
combined<-mergeDbSources(web_data,scopus_data,remove.duplicated=T)
##exporting file
write.xlsx(combined,"combinedabs.xlsx")
```
